# Theories of Sanitation

**Published:** 1894-11-17

**Authors:** 


### The First Century of English Medical Literature.

### X.—THEORIES OF SANITATION.

Manuals of health, dealing with prevention rather than cure, were exceedingly popular among the early reading public in England. They were largely modelled on the Regimen Sanitatis Salernæ, already mentioned, but were generally brought up to date by the addition of the author's own theories or experience. The cautions and counsels which they contain throw no little light on the social condition of our ancestors.

Boorde is well before his time in the stress which he lays on the necessity for pure air. In his list of those things which tend to corrupt the air, he begins where most men were content to end, at the influence of the stars. The "influenza" was a highly convenient explanation of any mysterious outbreak of infection, and served for some centuries to dispense with the necessity of hunting up a less remote cause. Boorde, however, believed in it only in a general way. He cautions his readers in particular against three remediable causes of corrupted air, viz., exhalations from marshes, carrion lying above ground, and "much people in a small room." There must be no stinking ditches, gutters, nor canals; no corrupt dunghills, nor sinks unless often cleaned. He has a special dread of dust, and directs no sweeping shall be done as long as any honest man is in the precinct of the house. This can be accounted for by the extraordinary foulness to which the floors were reduced by the accumulation of refuse from the table, &c., and was largely due to the habit of cleaning up only at stated intervals when the family departed, often fairly driven away by the state of the house, to another seat, a process commonly known, according to Miss Strickland, as "going to sweeten." Beware of emptying foul matter into the chimneys during summer, continues Boorde, a caution which speaks volumes. Beware also of the snuff of candles and the savour of apples, for these are contagious and infective. He would have all cesspools and sanitary accommodation placed at a distance from the house, a very necessary precaution in days when the cabinet led frequently directly out of the bedroom, from which every means was taken to exclude air. Boorde is no wiser than his age in his dread of night air, but he does provide for a certain amount of ventilation by ordering a fire in the bed-room night and morning "to dry up evil vapours."

It is difficult to account for the universal prejudice against night air which has pervaded all nations and all ages until close upon our own times. No single instance can be quoted of a medical writer rising superior to this curious delusion. The dread of cold water as a drink is a marked characteristic of all the olden authorities on health, and no feature of quackery was more severely condemned than an occasional eccentric recommendation of its value. The difficulty of obtaining it pure and unpolluted may partly account for the bad reputation of cold water. Boorde is decidedly of opinion that water, cool by itself, is not wholesome for an Englishmen; he recommends as about the best "running water, the which doth swiftly flow from east to west upon stones or pebbles." Sir William Vaughan, in his "Natural and Artificial Directions for Health," has also strong views on the water question. He deems it better on clay than on stone, and most wholesome in summer. Emphatically "it ought seldom to be drunk." If compelled to drink water, "see that you seeth it gently first." If putrid revive it with oil of sulphur or aqua vitæ. Perhaps the strongest anti-water authority is Thomas Cogan, headmaster of Manchester Grammar School, who wrote "The Haven of Health." He quotes an opinion of Eliot's that water if used for a child may be a harmless and even a sufficient drink; but he is very certain that men by nature are wine-drinkers, "except some odd abstemious, one among a thousand perchance, degenerate and of a doggish nature, for dogs of nature do abhor wine."

The best substitute for water, according to most writers on health, was ale. "Ale made of barleymeal and good water doth make a man strong." But even in those days, Sir William Vaughan complains, they had begun to adulterate by adding "slimy and heavy baggage unto it." An ordinary drink for sickness and health was made of barley, licoras, violet seed, parsley seed, red roses, sage, hyssop, figs, and raisins, boiled with water in an earthen vessel and set to cool. The poor, for whom this drink was too costly, were directed to gather tops of heath, whereof brushes are made, and dry them and brew a cheap drink, with a little licoras added if possible. In summer they were to drink vinegar and water. The Welsh drank metheglin, brewed from hot herbs, honey, and water. In many country places mead brewed from honey alone was the popular drink, while a richer compound, called Hippocras, was compounded of good claret or white wine mixed with cinnamon, ginger, and grains.

The rules of diet were exceedingly numerous and complicated. Every article of food had definite degrees assigned to it of heat and moisture, or the contraries. Most of the precepts in vogue were taken bodily from the ancients or from Continental writers, without heed to variations of climate or produce, and convey no notion of English customs.

The influence of clothing on health had hardly yet come to be recognised. Boorde gives the quaint counsel, "in winter next your shirt use to wear a peticote of scarlet," and would have the nightcap of scarlet also. The first suggestion of ventilated clothing is in the hint to cut a hole in the top of the nightcap, so that the hot vapours of the body may escape.

The abuse of drug-taking, against which these health manuals were aimed, was prevalent among all classes. Dr. Bullein complains that the public, "if they feel never so small or light a grief, they must presently to physick, until they have so filled their bodies with drinks that they are sicker of their physic than of any disease."

It is hardly likely that those on whom this passion had gained would be won from it by directions to bathe their heads four times a year, carry pomanders of sweet perfumes to make the heart merry, and use loud readings and disputations for the good of their breath. But these and such like simple counsels, with which the books of the period abound, were, at any rate, uniformly harmless, and often contained germs of wisdom not fully developed till the dawn of scientific hygiene.

